# Post-intubation tracheal stenosis: a rare clinical image

**DOI:** 10.11604/pamj.2024.47.135.42756

**Published:** 2024-03-22

**Authors:** Ashwin Karnan, Pankaj Wagh

**Affiliations:** 1 Department of Respiratory Medicine, Jawaharlal Nehru Medical College, Datta Meghe Institute of Higher Education and Research, Sawangi (Meghe), Wardha, Maharashtra, India

**Keywords:** Intubation, tracheostomy, stenosis, granulation, cough, dyspnoea

## Image in medicine

A 27-year-old male presented to the outpatient department with complaints of cough and breathlessness on exertion for the past 15 days. The patient gave a history of pneumonia 6 weeks back for which he was admitted, intubated in view of respiratory failure, treated with intravenous antibiotics, and extubated after 8 days. Post-discharge, the patient developed the above-mentioned complaints. Fibre optic flexible bronchoscopy showed significant tracheal stenosis (cuff type), biopsy taken which was suggestive of chronic granulation tissue with fibroblast infiltration. The trachea is a tubular structure that has a length of 10 to 12cm and a diameter of 22mm in males and 18mm in females. Tracheal stenosis following intubation or tracheostomy is a rare complication, but airway injury is commonly seen irrespective of the time period. High cuff pressure or prolonged intubation period may lead to ischemia and inflammation. Granulation tissue is formed with fibroblast proliferation, causing scarring and ultimately stenosis. Bronchoscopy is the mainstay of investigation. Treatment options include resection surgery, laser ablation, balloon dilatation, and stenting.

**Figure 1:**
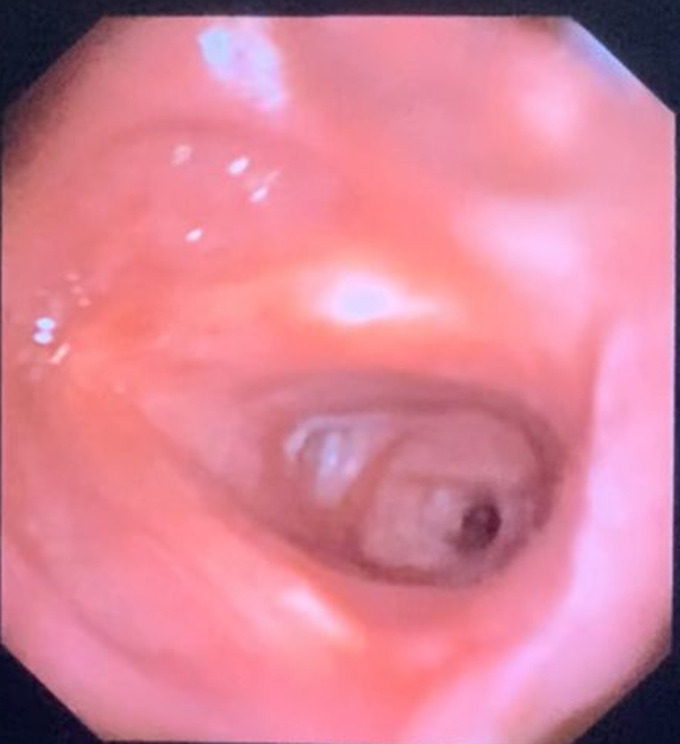
bronchoscopy image showing tracheal stenosis (cuff type)

